# Supplementary material for: An Experimental Study on Antioxidant Enzyme Gene Expression in Trematomus newnesi (Boulenger, 1902) Experimentally Exposed to Perfluoro-Octanoic Acid
Source: Antioxidants (Basel). 2023 Feb 2;12(2):352. doi: 10.3390/antiox12020352 (PMC9951861; doi:10.3390/antiox12020352)
Supplement: Supplementary file 1 [file antioxidants-12-00352-s001.zip › antioxidants-2168015-supplementary.pdf]

Table S1: Primer pairs used for qRT-PCR. Amplicon sizes and annealing temperatures (Ta) are also indicated.

isoform	PRIMER	Sequences 5' → 3'	Amplicon (bp)	T_a (°C)
sod1	SOD1_T.eulepidotus_FW	GCAAAGCTCAACATCACGGA	125	60
	SOD1_T.eulepidotus_RV	CCAGCATTGCCCGTCTTTAG		60
sod2	SOD2_T.bernacchii_FW	GTGGAAGCCTTCGTATCGCT	108	60
	SOD2_T.bernacchii_RV	GAAGGTAGTAGGCGTGCTCC		60
	7_SOD2_T.newnesi_FW	GCCTCAGCCAAACTTTAAACCTGG	135	60
	7_SOD2_T.newnesi_RV	CATGGTGCTTGCTGTGGTGC		60
gpx1	GPx1b_T.eulepidotus_FW	CCCTCCTCCCTCATGACTGA	145	60
	GPx1b_T.eulepidotus_RV	TGTCGCTGGTGAGGAACATC		60
gpx4	GPx4b_T.bernacchii_FW	TTTACGCATCCTCGCCTTCC	143	60
	GPx4b_T.bernacchii_RV	GATGAGCAGTGTCCCCGTTC		60
gapdh	GAPDH_T.bernacchii_FW	AAGTATGACTCCACCCACGG	113	60
	GAPDH_T.bernacchii_RV	ATGTTAGCGGGGTCCTTCTC		60
